# Design of splints based on the NiTi alloy for the correction of joint deformities in the fingers

**DOI:** 10.1186/1475-925X-9-49

**Published:** 2010-09-13

**Authors:** Sergio Puértolas, José M Pérez-García, Luis Gracia, José Cegoñino, Elena Ibarz, José A Puértolas, Antonio Herrera

**Affiliations:** 1 Department of Mechanical Engineering, University of Zaragoza, María de Luna 3, 50018 Zaragoza, Spain; 2 Department of Surgery, University of Zaragoza, Domingo Miral s/n, 50009 Zaragoza, Spain; 3 Department of Orthopaedic Surgery and Traumatology, Miguel Servet University Hospital, Paseo Isabel la Católica 1, 50009 Zaragoza, Spain; 4 Aragon Health Sciences Institute, Avenida Gómez Laguna, 25, 50009 Zaragoza, Spain; 5 Department of Science and Technology of Materials, University of Zaragoza, María de Luna 3, 50018 Zaragoza, Spain

## Abstract

**Background:**

The proximal interphalange joint (PIP) is fundamental for the functional nature of the hand. The contracture in flexion of the PIP, secondary to traumatisms or illnesses leads to an important functional loss. The use of correcting splints is the common procedure for treating this problem. Its functioning is based on the application of a small load and a prolonged stress which can be dynamic, static progressive or static serial.

It is important that the therapist has a splint available which can release a constant and sufficient force to correct the contracture in flexion. Nowadays NiTi is commonly used in bio-engineering, due to its superelastical characteristics. The experience of the authors in the design of other devices based on the NiTi alloy, makes it possible to carry out a new design in this work - the production of a finger splint for the treatment of the contracture in flexion of the PIP joint.

**Methods:**

Commercial orthosis have been characterized using a universal INSTRON 5565 machine. A computational simulation of the proposed design has been conducted, reproducing its performance and using a model "ad hoc" for the NiTi material. Once the parameters have been adjusted, the design is validated using the same type of test as those carried out on commercial orthosis.

**Results and Discussion:**

For commercial splint the recovering force falls to excessively low values as the angle increases. Angle curves for different lengths and thicknesses of the proposed design have been obtained, with a practically constant recovering force value over a wide range of angles that vary between 30° and 150° in every case. Then the whole treatment is possible with only one splint, and without the need of progressive replacements as the joint recovers.

**Conclusions:**

A new model of splint based on NiTi alloy has been designed, simulated and tested comparing its behaviour with two of the most regularly used splints. Its uses is recommended instead of other dynamic orthosis used in orthopaedics for the PIP joint. Besides, its extremely simple design, makes its manufacture and use on the part of the specialist easier.

## Background

The proximal interphalange joint (PIP) is fundamental for the functional nature of the hand. It is considered to be the functional epicentre, since 85% of total encompassment when an object is grasped depends on this joint [1]. The contracture in flexion of the PIP, secondary to traumatisms or illnesses leads to an important functional loss.

The use of correcting splints is the common procedure for treating this problem. Its functioning is based on the application of a small load and a prolonged stress which can be dynamic, static progressive or static serial [2]. Despite the force applied being small and progressive the neighbouring joints should be evaluated before its use in patients with systematic illnesses, as the splints increase the stress on the joints and can cause finger edema [3]. This progressive application of forces on the PIP joint stimulates the histic changes, which enable the elongation of the capsuloligamentous structures until the correction of the deformity is achieved [4].

The straightening forces developed by static splints were analysed by Wu [5] and those of the dynamic splints by Fess [6]. Both systems base the biomechanical action on the application of three parallel forces. Later analysis [3] consider that the force released by both systems is similar, and present the pressure exerted on the back of the damaged joint (PIP), greater in the static systems, as the main inconvenience of both [3].

The materials used in both types of splints are to a great extent thermoplastics, which in the short term suffer a change in resistance [7]; New materials such as neoprene have been proposed by other authors [8], although with this proposed model the inconvenience of covering all of the finger arises, something which can generate the edema and be counterproductive.

Considering the effectiveness of the two systems, good results have been published for both [9-12]. However it is fundamental to know the biomechanics of each system in order to produce personalised devices adapted to the characteristics of each patient [3].

It is important that the therapist has a splint available which can release a constant and sufficient force to correct the contracture in flexion. Nowadays NiTi is commonly used in bio-engineering, due to its superelastical characteristics and its shape memory [13]. The experience of the authors in the design of other devices based on the NiTi alloy [14-17], makes it possible to carry out the proposed design in this work - the production of a finger splint for the treatment of the contracture in flexion of the PIP joint.

This paper describes the characteristics of the splint designed, comparing its biomechanical behaviour with that of commercial dynamic splints regularly used to treat the stiffness of the PIP joint.

## Methods

The first step consisted in characterising the biomechanical properties of the splints that were to be used as reference. Two of the most regularly used splints have been chosen: the LMB Spring Finger Extension Splint (splint 1) and the LMB Spring-Coil Finger Extension Splint (splint 2).

**Figure 1:**
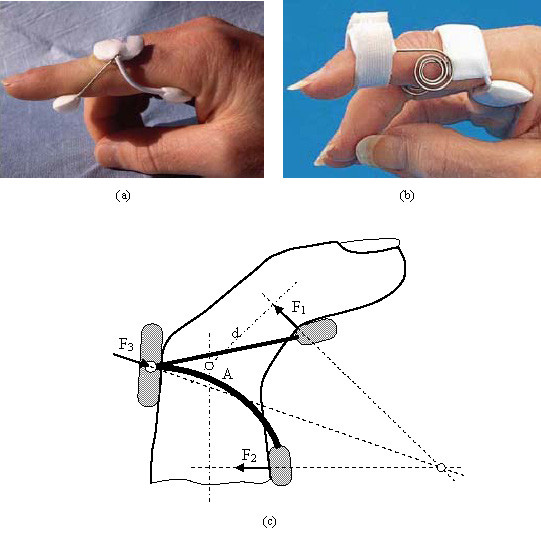
Bort type splints: a) LBM Spring Finger Extension Splint; b) LMB Spring-Coil Finger Extension Splint; c) Force transmission mechanism for Bort type splints

We are dealing with two simple designs which basically consist of a torsion spring with two angled arms which make it possible to fix and lock onto the finger (Figs. 1a and 1b). The spring restoration torque is the origin of the forces applied to the extremes of the splint, which are balanced with the reaction in the central section (Fig. 1c).

**Figure 2:**
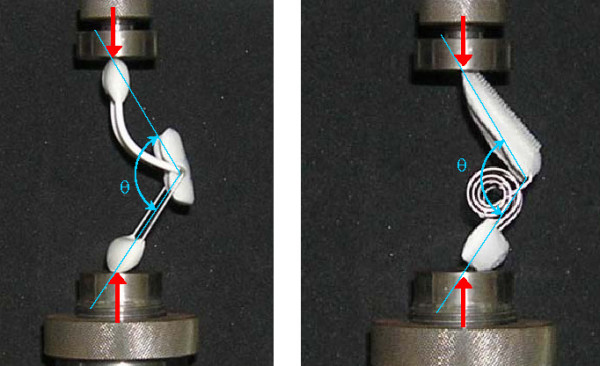
Bending test for Bort type splints

In order to characterise the recovering forces in the whole area in which the devices act, we proceeded to carry out the respective bending tests in an INSTRON 5565 universal test machine by means of eccentric compression of the orthosis (Fig. 2). This test was carried out instead of the 3-point bending test due to the fact that the 3-point test suffers from interferences between the actuators making it impossible to arrive at angles that are closed enough. Both tests were carried out with control in vertical movement at a speed of 2 mm/min. During the tests data for both force (N) and displacement (mm) were captured. Front view digital photos of the different positions of the extremes of the orthosis every 0.5 mm of vertical movement have also been captured. Later, an analysis of the photographs has been carried out using a computer program for image processing in order to obtain the variation of the angle between the extremes of the orthesis in the course of the tests. In this way, both the force-displacement graphs and the moment-angle graphs can be obtained.

**Figure 3:**
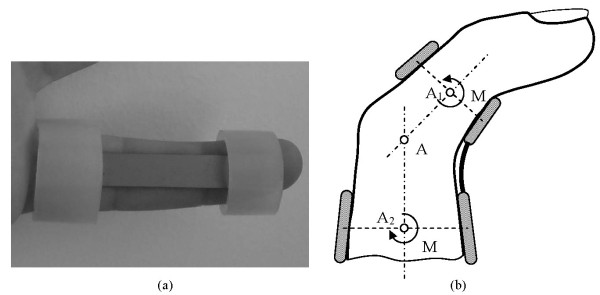
a) Prototype of the designed NiTi splint; b) Force transmission mechanism for the designed splint

**Figure 4:**
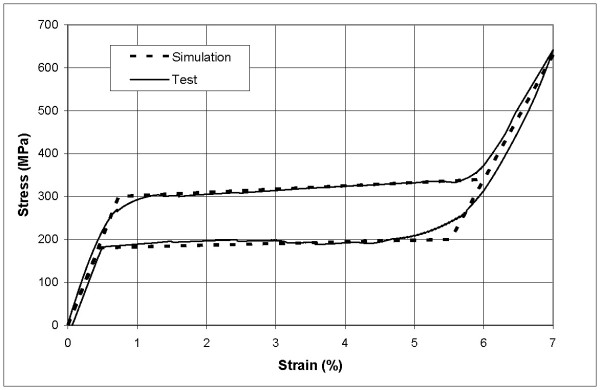
Stress-strain curve for loading and unloading process corresponding to the NiTi alloy at 22°C

The proposed design uses a thin plate of NiTi, which is fixed onto the finger by means of rings, which are responsible for transmitting the recovering force (Fig. 3). The mechanism provides a practically unidimensional bending performance, such that the device presents a mechanical response close to the intrinsic material behaviour. This means that the moment-angle curve of the splint has a similar shape to the material tension-deformation curve. This curve is characterized by means of a tensile test on the plate, carried out using the same INSTRON 5565 machine, observing a wide area of restoration at a practically constant tension, which is presented in Fig. 4. As far as the material is concerned, NiTi is an equiatomic alloy of nickel and titanium (commercially known as Nitinol), discovered in the U.S. Naval Ordenance Laboratory [18]. It belongs to a group of materials with shape memory (SMA). Basically, these alloys have the attribute of being able to recover a previously defined form when the material is subjected to an adequate thermal treatment; associated to this behaviour, the material has a super elasticity which lends to the property of withstanding large elastical deformations with relatively low tensions. This property is due to the change of phase austenite-martensite-austenite which the material undergoes when it is subjected to tension [19].

The action of the splints is directly related to the rigidity, and in the proposed design the rigidity is directly related to the width, the thickness and the length, although all of these geometric factors work in an uneven way. An increase in width supposes a linear growth in the recovering force and a better finger support. However, the most important factor used to control the force exerted by the splint is the plate thickness. The device is very sensitive to thickness change, presenting a cubic rate influence. Hence, the greater the thickness, the greater the effect of straightening and the smaller the risk of rupture although it is more difficult to bend the splint and fit it in the volar zone of the injured finger. On the contrary, if the thickness is reduced so is the straightening effect and the risk of rupture increases, although it is easier to bend the splint and fit it on the finger

To obtain a design which transmits a force adequate for the recovery of the original position of the finger, a simulation by means of finite elements for a plate of these dimensions 80 × 10 × 1 mm is carried out equivalent to the test undertaken for commercial splints. For the behaviour of the material a proprietary developed user subroutine is used, based on Auricchio`s models [19], in the Abaqus program [20], after previously carrying out an adjustment of parameters from the results of the tensile test (Fig. 4). In Table 1 the different parameters used in the simulation are gathered. The model consists of 4455 nodes and 3200 hexaedric elements, type C3D8, with linear approximation (80 in length, 10 in width and 4 in thickness). As for the boundary conditions, initially a displacement of 1 mm in the centre of the plate is applied to later apply the eccentric compression until reaching the maximum curvature, moment in which the load is removed and a free restoration is produced.

**Table 1:** Material properties (NiTi)

Parameter	Description	Value
*E*_*A*_	Austenite Young Modulus	52650 MPa

*ν*_*A*_	Austenite Poisson Ratio	0.33

*E*_*M*_	Martensite Young Modulus	38250 MPa

*ν*_*M*_	Martensite Poisson Ratio	0.33

*ε*_*L*_	Maximum Transformation Strain	6%

$\sigma_{s}^{AM}$	Transformation Activation Stress (A→M)	300 MPa

$\sigma_{c}^{AM}$	Transformation Completion Stress (A→M)	340 MPa

$\sigma_{s}^{MA}$	Transformation Activation Stress (M→A)	200 MPa

$\sigma_{c}^{MA}$	Transformation Completion Stress (M→A)	180 MPa

*T*_0_	Reference Temperature	22°C

*C*^*AM*^	$\frac{\partial \sigma_{s,c}^{AM}}{\partial T}$	6.7 MPa/°C

*C*^*MA*^	$\frac{\partial \sigma_{s,c}^{MA}}{\partial T}$	6.7 MPa/°C

**Figure 5:**
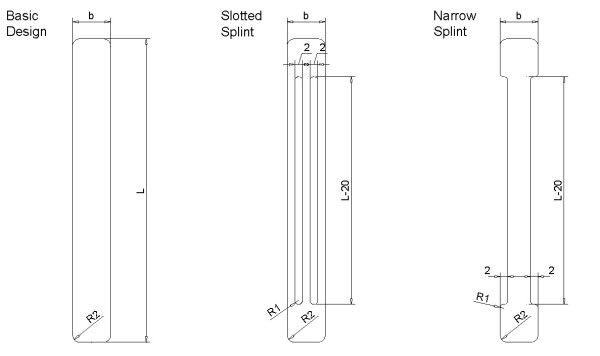
Alternative designs for the proposed splint

The results of this first simulation applied recovering forces well above those corresponding to the commercial splints that were analysed, so we proceeded to review the initial design, either by adjusting the thickness of the plate or looking for alternative designs as those shown in Fig. 5. With any one of the three proposed designs it is possible to carry out an adjustment to the parameters in order to obtain recovering forces within the desired range.

Finally, we proceeded to produce prototypes of the proposed splint, by means of mechanized electroerosion, from plates with 1 mm of NiTi thickness (50.8 at %Ni, 49.2 at % Ti), supplied by the company Memory Metalle GMBH (Germany). The plates with less thickness (up to 0.5 mm) were obtained by cold lamination of the cut plates, followed by a annealing to eliminate the effects of cold lamination. Tests were then carried on these prototypes equivalent to those done on the commercial splints analysed, following the same procedure as the one described for the simulation.

## Results

**Figure 6:**
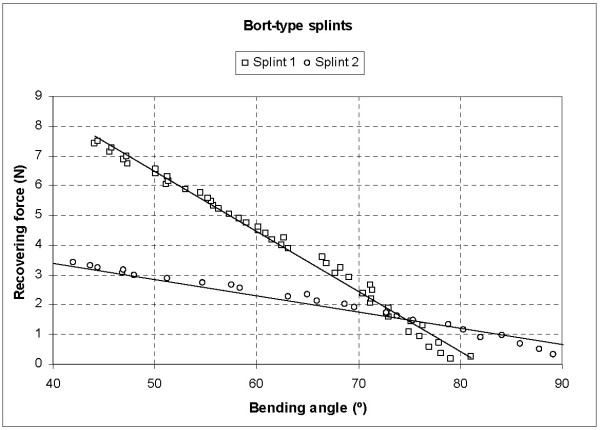
Recovering force-Angle curves for commercial Bort type splints (tests)

The results obtained in the tests carried out on commercial splints are presented first of all. In Fig. 6 we can see the Recovering Force-Angle curves for both splints. The behaviour is practically linear in both cases, with the following curve fittings (Eq. (1) and Eq. (2)):

(1) $$-\text{ Splint 1:}\;\text{ F}=-0.\text{2}0\text{19}\theta +\text{16}.\text{582},{\text{R}}^{\text{2}}=0.\text{99}0$$

(2) $$-\text{Splint 2}:\;\text{ F}=-0.0\text{545}\theta +\text{5}.\text{554},{\text{ R}}^{\text{2}}=0.\text{985}$$

The analysis of the slopes of these straight lines makes it possible to determine the rigidity to bending for each case. For the first splint, a slope of 0.2019 is obtained, whilst the second has a slope of 0.0545. The recovering force falls to excessively low values in both splints as the angle increases.

**Figure 7:**
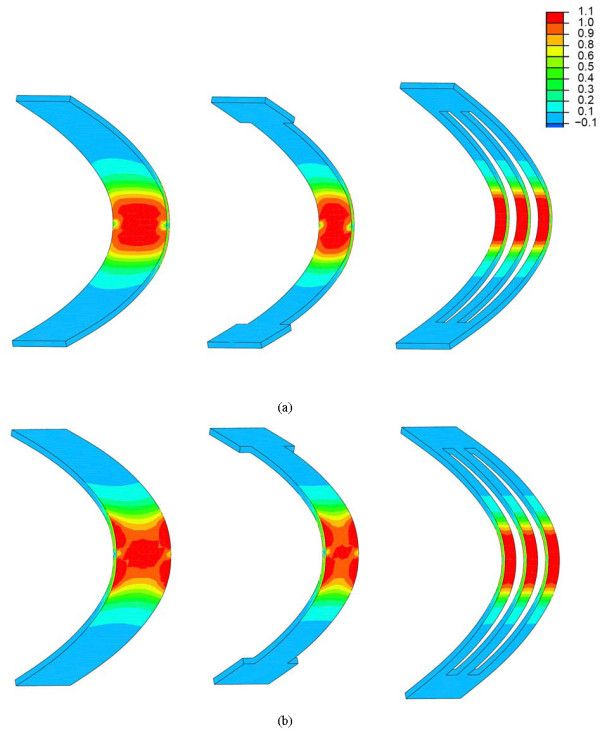
Martensite fraction: (a) compression side; (b) tension side

In Fig. 7 we can see the austenite-martensite transformation maps, showing the fraction of martensite produced in each case, induced by the tensional level reached. A generalized phase transformation in the central section of the plate can be seen. This is essential in order to achieve a controlled recovering force.

**Figure 8:**
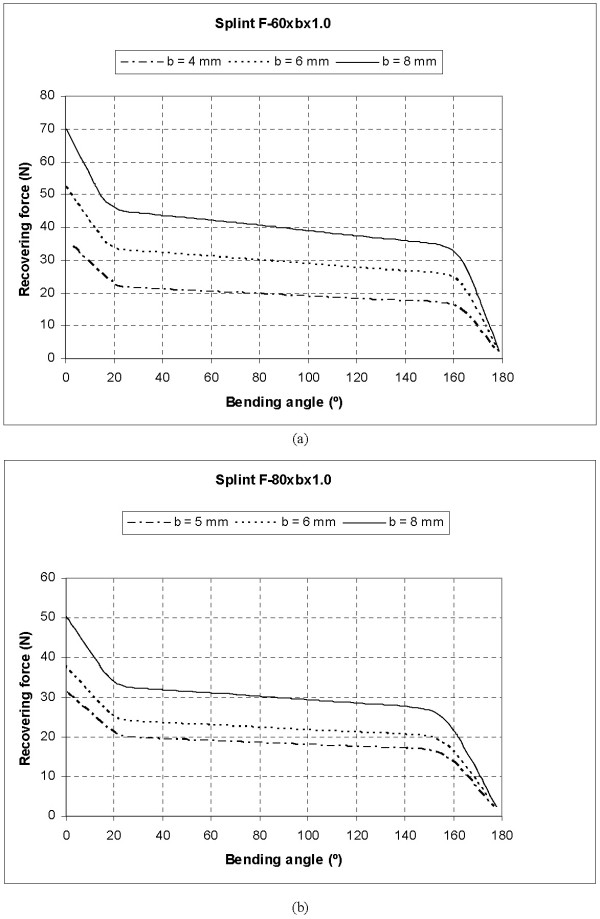
Recovering force-Angle curves: (a) Length 60.0 mm, width variable, thickness 1.0 mm; (b) Length 80.0 mm, width variable, thickness 1.0 mm

**Figure 9:**
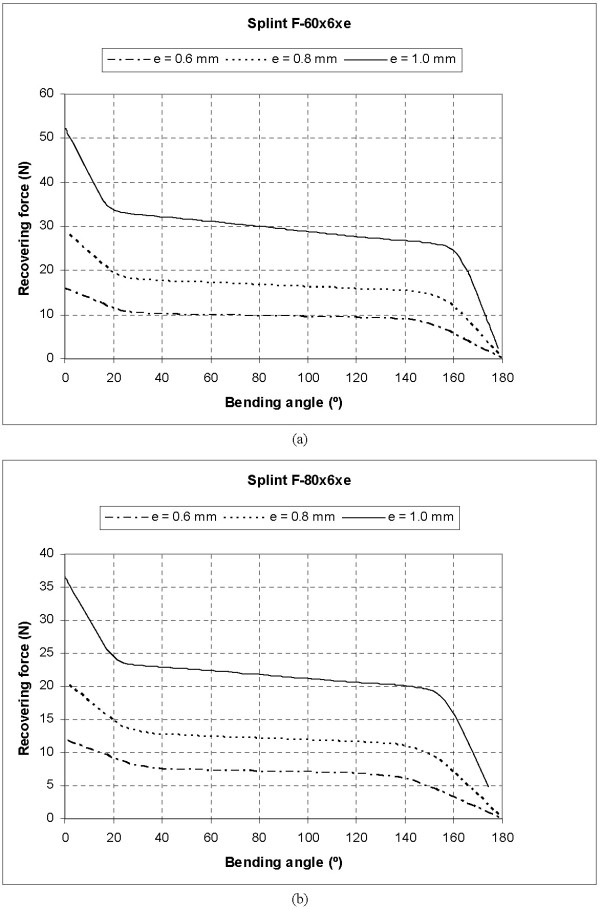
Recovering force-Angle curves: (a) Length 60.0 mm, width 6.0 mm, thickness variable; (b) Length 80.0 mm, width 6.0 mm, thickness variable

Fig. 8 shows the Recovering Force-Angle curves for the different lengths and widths of the plate, with a fixed thickness of 1 mm, while in Fig. 9 we can see the Recovering Force-Angle curves for different lengths and thicknesses of the plate, with a fixed width of 6 mm. In these a practically constant recovering force value can be observed over a wide range of angles that vary between 30° and 150°. This makes it possible to define a characteristic value of recovering force which is ascribed to an angle flexion of 80°. The same occurs in all of the cases analysed. A summary of this can be seen in Table 2, indicating the characteristic ranks of recovering force obtained.

**Table 2:** Splint sizes and recovering forces

Designation	Length (mm)	Net width (mm)	Thickness (mm)	Characteristic Recovering Force (N)				
				
				5≤F<10	10≤F<15	15≤F<20	20≤F<25	25≤F≤30
F-80 × 6 × 0.6	80	6	0.6	7.0				
F-70 × 6 × 0.6	70	6	0.6	8.0				
F-40 × 6 × 0.4	40	6	0.4	7.5				

F-80 × 6 × 0.8	80	6	0.8		12.5			
F-80 × 4 × 1.0	80	4	1.0		14.0			
F-70 × 6 × 0.8	70	6	0.8		13.8			
F-60 × 6 × 0.6	60	6	0.6		10.0			
F-40 × 6 × 0.5	40	6	0.5		11.5			

F-80 × 5 × 1.0	80	5	1.0			18.0		
F-60 × 6 × 0.8	60	6	0.8			16.5		
F-40 × 6 × 0.6	40	6	0.6			16.0		

F-80 × 6 × 1.0	80	6	1.0				23.0	
F-60 × 4 × 1.0	60	4	1.0				20.0	

F-80 × 8 × 1.0	80	8	1.0					29.0
F-70 × 6 × 1.0	70	6	1.0					28.5
F-60 × 6 × 1.0	60	6	1.0					30.0

**Figure 10:**
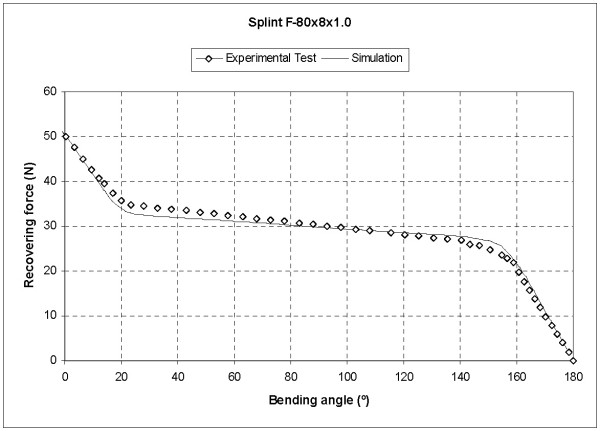
**Recovering force-Angle curves for the 80 × 8 × 1.0 mm splint**. Experimental test versus simulation

Finally, in order to verify the simulation results an experimental bending test was carried out on a prototype made in accordance to the developed design, obtaining the results shown in Fig. 10. A perfect agreement between the simulation results and those obtained in the experimental bending test can be seen, except in the phase transition zones (austenite-martensite, at the begining of the plateau; martensite-austenite, at the end of the plateau). The difference is due to the linearization of strain-stress curve in such zones used (Fig. 4) in the simulation.

## Discussion

Although good therapeutic results have been published for the use of dynamic and static systems [9-12], tests have been carried out on two regularly used dynamic models, in order to obtain information about the mechanical behaviour.

From the results obtained it was deduced that the bending behaviour of the two commercial prostheses analysed is practically linear, due to the behaviour of the torsion spring. The recovering forces are in maximum values around 7.5 N and 3.5 N, respectively, for an angle of 40°. These forces suffer a notable decrease as the angle increases (recovering), reaching values that can be insufficient for the recuperation of the joint along an angle of about 30°, and with values that are practically null from 85-90° (Fig. 6). This makes it necessary to plan a therapy in phases, with the progressive substitution of splints, adjusting the rigidity in each phase. The main thing is that the therapist has a splint at his/her disposal capable of releasing a constant and sufficient force which is able to correct the contracture in flexion and assure a good final result [3].

In order to design a NiTi prosthesis with a recovering force equivalent to that of a Bort orthosis, it is necessary to adjust the rigidity to bending to the value of this. A parametric simulation on three alternative designs has been carried out, covering a range of forces from 5 to 30 N, with lengths and widths capable of adapting to fingers of different sizes. One main advantage of the proposed designs is that they achieve a practically constant recovering force in a range of angles from 20° to 150°. This makes one therapeutic stage possible that practically covers the total recovery of the PIP joint.

Apart from the properties of NiTi, the biomechanical behaviour of the proposed prosthesis compared to the commercial ones analysed is totally different due to its design. Hence, the mechanism to transmit forces onto the finger in the Bort class of splints (and in the majority of those that exist on the market) is based on equilibrium in a simple bending situation, with action (F_1_, F_2_) and reaction (F_3_) in the different parts of the splint (Fig. 1c). On the contrary, in the developed prototype the transmission mechanism is based on equilibrium in pure bending, transmitting both torques on the fixation rings (Fig. 3b), through the local equilibrium of forces in the fixation rings.

The common bending mechanism has two important drawbacks: firstly, for a linear behaviour spring the ratio between recovering force and angle is constant in its whole length. Hence, as recovery is produced the torque transmitted on the joint decreases significantly, even though the distance from the point where the force is applied F_1 _on the joint, point A in Fig. 1c becomes progressively larger. Its increase does not compensate the loss of force, for which it is necessary to change the splint for another with a different force calibration. Moreover, the forces involved in the equilibrium have components that can generate compression or traction on the joint itself, possibly increasing the damage to the joint.

However, in the mechanism of pure bending as it is directly transmitting torques, the effect on the joint is always the same in all of the recovering range. In addition forces are not generated on joints; the forces are generated at local level on the fixation rings to give rise to the torques transmitted, acting on zones that are away from the joint and without damaging effects. Thus the recovering moment is constant in all of the length A_1_-A-A_2 _(Fig. 3b), without producing undesired effects on the joint. Given that the splint reproduces in the graph moment-angle the basic behaviour of NiTi in the tensile test, the recovering moment is practically constant in the all of the range where the splint works. According to [4,21] a prolongated constant stress is a key factor achieving soft tissue remodeling; our model of splint offers the advantages to adjust the recovering force depending on the dimensions on the sheet, producing a constant recovering force along a wide range of splinting. Then the whole treatment is possible with only one splint, and without the need of progressive replacements as the joint recovers.

No other splint available on the market offers this property since all of them are based on materials and mechanisms whose global result is that of a cuasi-linear behaviour. This makes it impossible to obtain the curves with a practically null slope in the recovery stage like the one presented here.

Another talking point is the optimum time for the use of straightening splints, which Flower defined as TERT (Total End Range Time) [22], having checked that the longer it is worn daily the better the results [22,23]. However, Flower himself concludes that in addition to the time of use, the force application parameters are fundamental in attaining a good correction of the deformity [23]. Due to its comfort the proposed design makes it possible to wear permanently. On the other hand, permanent action dynamic orthosis, regularly used in orthopaedics for the PIP joint, are difficult to fit, above all at the level of the proximal phalange despite the therapist being able to choose the size.

On the contrary, the designed splints in this work improve the initial adjustment and make it easier to use for both the patient and the specialist, without presenting difficulties in its fitting. The proposed design also avoids the harmful effect of pressure on the back of the joint [3] which is produced in the usual static and dynamic systems.

## Conclusions

A new model of splint has been designed, simulated ant tested comparing its behaviour with two of the most regularly used splints. The advantages offered against the most frequently used commercial models can be summarised as:

- Better control of the recovering force.

- Maintaining the splint over long periods of time without replacement, since its effect remains unalterable over a wide period of recuperation.

- Ease of use for both the patient and specialist.

- Ease of producing made to measure designs for each patient.

- Significant economic saving in the treatment.

For all of the above mentioned, the proposed design is highly competitive compared to those used presently. In fact, our initial clinical trials have confirmed the biomechanical advantages of the proposed design.

## Competing interests

The authors declare that they have no competing interests.

## Authors' contributions

JPG and AH carried out the design of the splint. SP, LG and EI carried out the finite element simulations. JC and JP carried out the experimental tests. All authors were involved in the study design and writing of the manuscript. All authors read and approved the final version of the manuscript.

## References

[B1] Prosser R Splinting in the management of proximal interphalangeal joint flexion contracture Jour Hand Therapy 1996 9 378 386 10.1016/s0894-1130(96)80045-7 8994014

[B2] Wilton J Saunders WB Hand splinting: Principles of design and fabrication 1997 London

[B3] Li C Force analysis of the Belly Gutter and Capener splints Jour Hand Therapy 1999 12 337 343 10.1016/s0894-1130(99)80074-x 10622202

[B4] Fess EE McCollum M The influence of splinting on healing tissues Jour Hand Therapy 1998 11 157 161 10.1016/s0894-1130(98)80014-8 9602973

[B5] Wu SH A belly gunter splint for proximal interphalangeal joint flexion contracture Amer Jour Occup Therapy 1990 45 939 943 10.5014/ajot.45.9.839 1928292

[B6] Fess EE Force magnitude of commercial spring-coil and sring-wire splint designed to extend the proximal interphalangeal joint Jour Hand Therapy 1988 1 86 90

[B7] Sheehan JL Winzeler-Mercay U Mudie MH A randomized controlled pilot study to obtain the best estimate of the size of the effect of a termoplastic resting splint on spasticity in the stroke-affected wrist and fingers Clin Rehabilitation 2006 20 12 1032 1037 10.1177/0269215506071267 17148514

[B8] Punsola-Izard V Rouzaud JC Thomas D García-Elías L Le collage en tension dans les orthèses dynamiques en matériau neoprene Chirugie de la Main 2001 20 231 235 10.1016/S1297-3203(01)00039-7 11496610

[B9] Benaglia PG Sartorio F Franchignoni F A new thermoplastic splint for proximal interphalangeal joint flexion contractures Jour Sports Med Phys Fitness 1999 39 249 252 10573669

[B10] Li-Tsang CWP Hung LK Mask AFT The effect of corrective splinting on flexion contracture of rheumatoid fingers Jour Hand Therapy 2002 15 185 191 10.1053/hanthe.2002.v15.015018 12086029

[B11] Glasgow C Wilton J Tooth L Optimal daily total end range time for contracture: resolution in hand splinting Jour Hand Therapy 2003 16 3 207 218 10.1016/S0894-1130(03)00036-X 12943123

[B12] Schwartz DA Janssen RG Static progressive splint for composite flexion Jour Hand Therapy 2005 18 447 449 10.1197/j.jht.2005.07.005 16271693

[B13] Petrini L Migliavacca F Massarotti P Schievano S Dubini G Auricchio F Computational studies of shape memory alloy behavior in biomedical applications Jour Biomech Eng 2005 127 4 716 725 10.1115/1.1934203 16121543

[B14] Puértolas JA Pérez-García JM Juan E Rios R Design of a suture anchor based on the superelasticity of the Ni-Ti alloy Biomed Mater Eng 2002 12 3 283 289 12446943

[B15] Lahoz R Gracia L Puértolas JA Training of the two-way shape memory eggect by bending in NiTi alloys Jour Eng Mater And Technology 2002 124 4 397 401 10.1115/1.1495001

[B16] Domingo S Puértolas S Gracia L Mainar M Usón J Puértolas JA Design, manufacture and evaluation of a NiTi stent for colon obstruction Bio-Med Mater Eng 2005 15 5 357 365 16179756

[B17] Domingo S Puértolas S Gracia L Puértolas JA Mechanical comparative analysis of stents for colorectal obstruction," Min Invas Ther And Allied Technologies 2006 15 6 331 338 10.1080/13645700601037954 17474056

[B18] Buehler WJ Wiley RL Nickel-base alloys 1965 U.S. Patent 3.174.851

[B19] Auricchio F Petrini L Improvements and algorithmical considerations on a recent three-dimensional model describing stress-induced solid phase transformations Int Jour Num Methods in Engineering 2002 55 1255 1284 10.1002/nme.619

[B20] ABAQUS 2009 http://www.simulia.com/

[B21] Fess EE A history of splinting: To understand the presen, view the past Jour Hand Therapy 2002 15 97 132 10.1053/hanthe.2002.v15.0150091 12086034

[B22] Flower KR LaStayo P Effect of total end range time on improving passive range of motion Jour Hand Therapy 1994 7 150 157 10.1016/s0894-1130(12)80056-1 7951706

[B23] Flower KR A proposed decision hierarchy for splinting the stiff joint, with an emphasis on force application parameters Jour Hand Therapy 2002 15 158 162 10.1053/hanthe.2002.v15.015015 12086026

